# ESPO AND SOCIAL RESEARCH, POLICY, AND PRACTICE SECTION SYMPOSIUM: GOING THE EXTRA MILE: HOW PIONEERING GERONTOLOGISTS TRANSLATE RESEARCH TO POLICY AND PRACTICE

**DOI:** 10.1093/geroni/igae098.1025

**Published:** 2024-12-31

**Authors:** Taylor Jansen, Sara Bybee, Jennifer Greenfield

**Affiliations:** Chair; Co-Chair; Discussant

## Abstract

Gerontologists must have fortitude to take the extra step in translating research to end-users like community stakeholders, policymakers, and older adults; Otherwise, research can be futile. In this symposium, participants will hear from speakers who have engaged in translational research from the local to federal levels. Dr. Kina White, from the Mississippi State Department of Health will speak to her successful dissemination efforts informing state policymakers and health professionals of recent research conducted on older Mississippians. Dr. White personally mailed a copy of the research to each mayor in Mississippi, an excellent example of fortitude. Another state-level example will come from Dr. Howard Degenholtz, a Professor at the University of Pittsburg, who recently worked with the Pennsylvania Department of Aging to facilitate a statewide needs assessment survey using the Age Friendly Communities Framework, which helped develop a 10-year plan for the Pennsylvania Department of Aging. Finally, Dr. Sheryl Zimmerman, University of North Carolina (UNC) Distinguished Professor and Executive Director of the national Center for Excellence in Assisted Living (CEAL@UNC) will discuss CEAL’s role to advance excellence through stronger connections between research, practice, and policy, and related advocacy at the federal level. Overall, the aim of this symposium is to answer the “so what” question that many students and researchers are presented with and motivate gerontologists to broaden their dissemination and advocacy efforts to have the most positive impact on our society, whether in research, policy, or practice.

